# Risk factors and outcomes in asymmetrical femoral component size for posterior referencing bilateral total knee arthroplasty: a matched pair analysis

**DOI:** 10.1186/s12891-018-2220-6

**Published:** 2018-08-16

**Authors:** Piya Pinsornsak, Adisai Chaiwuttisak, Krit Boontanapibul

**Affiliations:** 1 Department of Orthopaedics, Faculty of Medicine, Thammasat University, 99 Moo 18, Khlong Nueng, Khlong Luang, Pathum Thani, 12120 Thailand; 2 Department of Orthopaedics, Chulabhorn International College of Medicine, Thammasat University, 99 Moo 18, Khlong Nueng, Khlong Luang, Pathum Thani, 12120 Thailand

**Keywords:** Bilateral total knee arthroplasty, Femoral component, Asymmetrical femoral component size, Anterior femoral offset, Posterior femoral offset, Femoral component flexion

## Abstract

**Background:**

Theoretically, potential errors in femoral component (FC) sizing can affect postoperative functional outcomes after total knee arthroplasty (TKA), including range of motion (ROM), anterior knee pain, and flexion stability. Incidences of asymmetrical femoral components (AFC) in bilateral TKA have been reported; however; there is a lack of data on exactly why AFC size selection may differ in patients who have had posterior referencing system bilateral TKA. Therefore, this study was conducted to determine risk factors of AFC size selection in patients specifically undergoing posterior referencing bilateral TKA and to compare clinical outcomes between those with AFC or symmetrical femoral component (SFC) sizes.

**Methods:**

We conducted a retrospective matched-pair study comparing thirty-four patients who had undergone simultaneous and staged bilateral TKA using AFC size (Group I) and thirty-five patients with SFC size (Group II). Patients were matched according to gender, body mass index, prosthesis type, and operative technique. Preoperative radiographic morphology of both distal femurs including anteroposterior/mediolateral diameters, anterior-posterior femoral offset, and postoperative radiographic data of FC comprising flexion and valgus angle were recorded. The postoperative functional outcomes including ROM, anterior knee pain, knee society score, and functional score at 6 weeks, 3, 6, 12 and 24 months were compared.

**Results:**

There were no differences in morphology between left and right distal femurs from preoperative radiographic data in both groups. The postoperative radiograph showed a significantly greater FC flexion angle difference in Group I vs. Group II (2.18° ± 1.29° and 1.36° ± 1.08° *P* = 0.007), while the other parameters were the same. The postoperative clinical outcomes displayed no distinction between groups.

**Conclusion:**

The factor primarily associated with AFC size selection in bilateral TKAs is the difference in FC flexion angle but not the morphological diversity between sides. The postoperative functional outcomes were not inferior in AFC patients in comparison with SFC patients.

## Background

The number of patients undergoing Total Knee Arthroplasty (TKA) has mirrored the growth in aging populations; some 20 % of elderly patients need bilateral TKA [1]. The proper choice of component size is thought to be essential for a good clinical outcome [2].

Theoretically, the femoral component size affects the flexion gap, stability, range of motion (ROM) and functional outcome after surgery. If the selected component is too small, the result could be flexion instability and pain, recurrent effusion, cam jump and dislocation in a posterior-stabilised prosthesis, and premature loosening of the component itself [3]. Conversely, too large of a femoral component can limit the ROM, create a painful and stiff knee, lead to anterior knee pain with patellar overstuff, and result in a poor functional outcome [4, 5]. In the mediolateral (ML) plane, too small of a component creates an under hang which may result in subsiding of the component, increased bleeding from the raw surface, and, finally, osteolysis [6] whilst too large of a femoral component enhances component overhang and may increase knee pain [5, 7].

An overview of previously published work shows that 7–9.2%of patients who had undergone a bilateral TKA had an asymmetrical femoral component (AFC) [8–10]. Asymmetrical incidences for anterior referenced femoral component were significantly higher than those using the posterior referencing system. This may be because of the irreproducibility of the flexion gap which will possibly create variability in femoral component sizing [9]. Many factors can influence AFC size selection, including asymmetrical patient anatomy between the left and right knees, the ligament laxity or tightness, the thickness of distal femoral cut which affects the extension gap, errors in distal femoral cutting angle, and the potential variability of the different anatomical landmarks used to measure (between surgeons) over the anterior surface of distal femur [11]. Overall, though, we consider that there is a lack of data on exactly why specific AFC sizes are chosen for patients using posterior referencing bilateral TKA. Therefore, the primary objective of this study was to determine the risk factors affecting AFC size selection for patients undergoing posterior referencing bilateral TKA, including the preoperative patient’s anatomy on both sides of the knee and the position of prosthesis component placement in sagittal and coronal plane. The secondary objective was to compare the clinical outcomes of patients who underwent posterior referencing bilateral TKA between AFC or SFC.

## Methods

### Study design and participants

For our retrospective review, we had 374 cases of bilateral TKA that were operated on, between March 2012 and June 2015, by a single surgeon (PP). We included all varus gonarthrosis patients classified with Kellgren-Lawrence Grade 3 to 4 who underwent either simultaneous bilateral (operation on both sides with the same anaesthesia) or staged bilateral (each side operated on independently with separate anaesthesia and admission) TKA. We excluded patients with previous knee injuries, deformed bone anatomy, post-traumatic knee arthritis, extra-articular knee deformity, inflammatory arthritis, and other deformities classified as severe (preoperative varus deformity > 20°, limited knee flexion < 90°, and flexion contracture > 20°). Inadequate preoperative and postoperative radiographs, including improper exposure, position and techniques affecting radiographic measurement, were also excluded. Therefore, we finally enrolled 319 patients from the original total of 374 bilateral TKA patients.

Among these cases, we identified thirty-five patients with AFC (Group I), and all had cemented posterior-stabilized total knee prosthesis (Vanguard® Knee System, Zimmer Biomet, Warsaw, Indiana, USA) using the posterior referencing system. SFC patients (Group II) were then place into matched pair in a 1:1 fashion. To do this, we selected 35 SFC from the total of 284 SFC bilateral TKA patients; this was based on gender, age at bilateral TKA (performed within a range of 5 years), body mass index [BMI (within 5 kg/m^2^)], prosthesis type, and operative technique. This study was approved by our institutional ethics committee.

### Sample size

We calculated sample size based on our pilot study. We estimated that a sample size of at least 33 patients in each treatment group would have 80% power to detect a mean femoral component size difference of at least 0.8 mm for the asymmetrical size group compared with the symmetrical size group, assuming an SD of 0.21, with a 5% one-sided type I error. We rounded this up to 35 patients in each group.

### Operative procedure

A standard medial parapatellar approach was used on all patients. Femoral preparation was performed first by drilling the insertion point of the femoral step reamer 1 cm above the posterior cruciate ligament insertion. After the intramedullary drill guide diameter 9 mm was inserted, the distal femoral cut was carried out with the valgus angle perpendicular to the mechanical axis, measured from the whole leg standing posteroanterior weight-bearing radiograph. Anterior cruciate and posterior cruciate ligaments were removed. The tibial extramedullary guide system was applied, and proximal tibial cut was made with a posterior slope aiming for 3 degrees and perpendicular to the mechanical axis. Ligament balancing to create a rectangular extension gap was performed and checked with a spacer block. Lower extremity alignment was measured, and the valgus-varus stability of knee was tested in full extension. The posterior referencing system for the AP cut was chosen. The femoral anteroposterior (AP) cut using the AP cutting guide was inserted with a 3-degree external rotation from the posterior condyle. The AP sizing was measured using an anterior boom position at the highest point of the anterolateral femoral cortex. In-between femoral component size was determined by using the closest size of the component. After finishing the AP cut, the flexion gap was balanced and checked with the spacer block in 90-degree knee flexion. The cemented posterior-stabilized total knee prosthesis (Vanguard® Knee System, Zimmer Biomet, Warsaw, Indiana, USA) was inserted, and the patella was resurfaced by restoring of natural patella thickness. Standard postoperative pain control and rehabilitation protocols were employed in all cases.

### Outcome measures

**Fig. 1:**
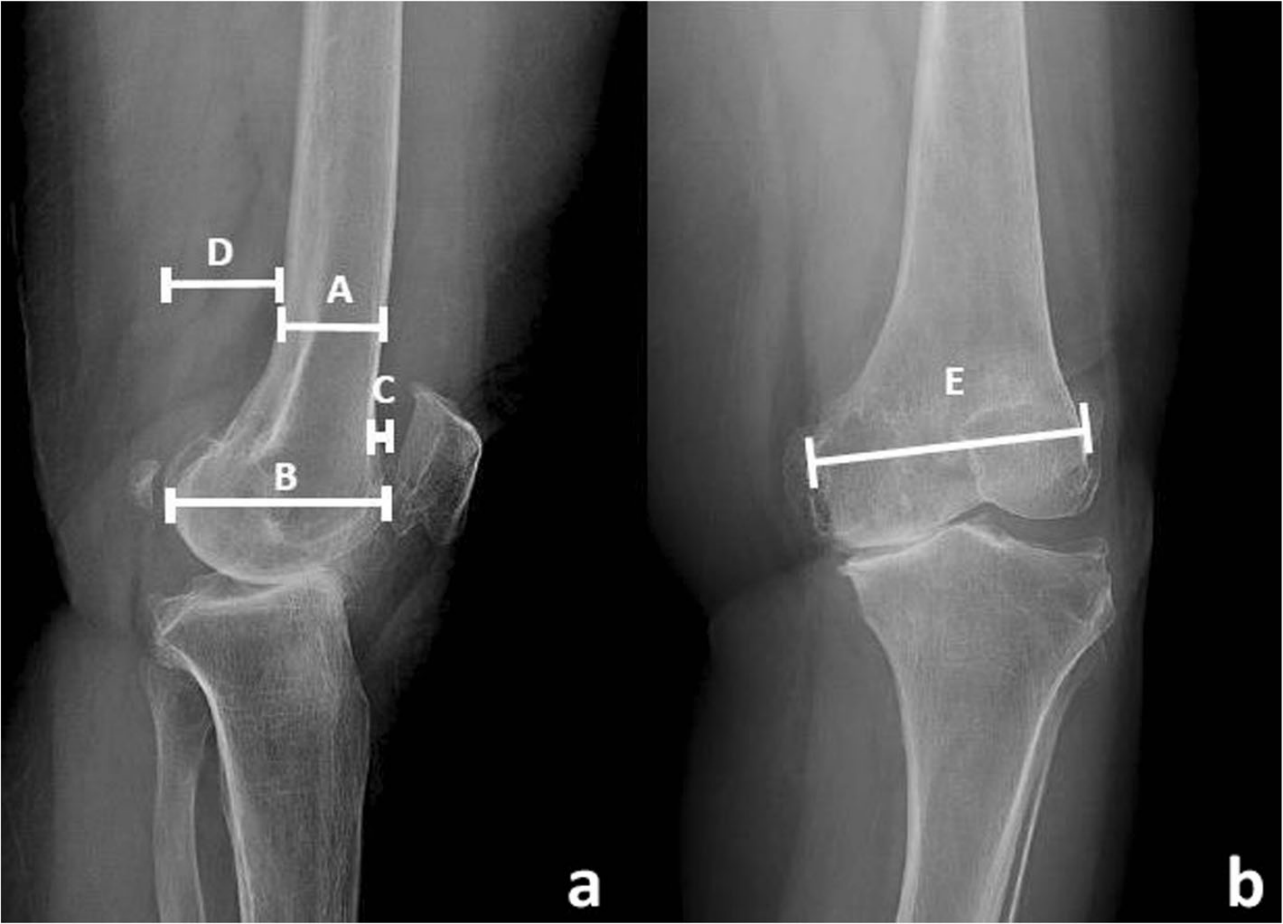
Preoperative radiographic measurements of distal femoral morphology. **a**: Measurement technique of diameter of AP femoral canal (A), diameter of AP femoral condyle (B), anterior femoral offset (C) and posterior femoral offset (D) in lateral view. **b**: Measurement technique of ML diameter of femoral condyle in AP view (E)

**Fig. 2:**
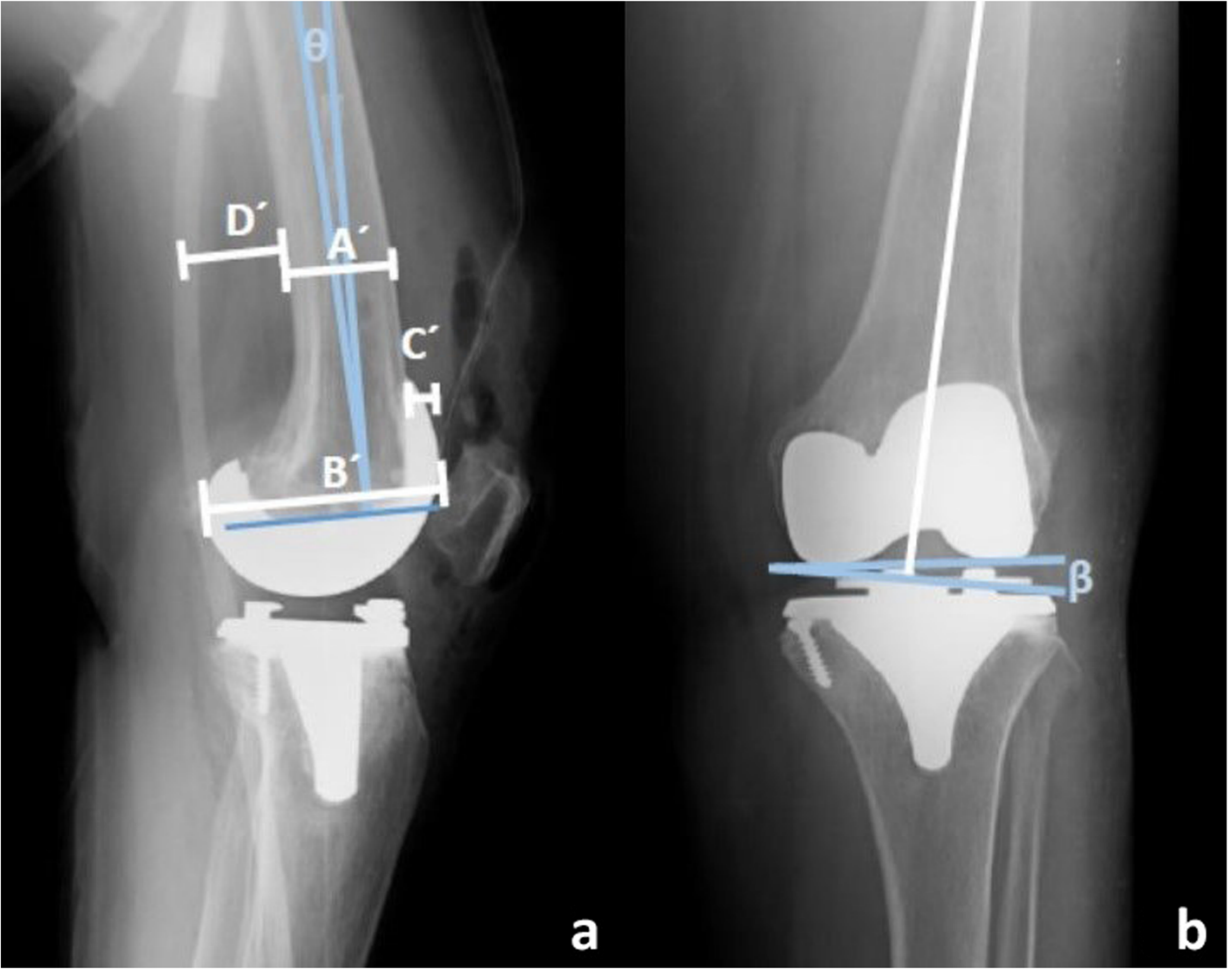
Postoperative radiographic measurements of femoral component. **a**: Measurement technique of diameter of AP femoral canal (A’), diameter of AP femoral condyle (B’), anterior femoral offset (C’), posterior femoral offset (D’) and femoral component flexion angle (ө) in lateral view. **b**: Measurement technique of femoral valgus angle (β)

The primary outcome measure was the preoperative evaluation of patient’s anatomy on both sides of distal femurs including the AP and ML diameters of the femoral condyle, the AP diameter of femoral canal, and the anterior and posterior femoral offset (Fig. 1). We also examined the postoperative radiographs to evaluate the femoral component flexion angle, femoral component valgus angle, the anterior and posterior femoral offset of the component, AP diameter of femoral canal, and AP and ML diameters of the femoral component (Fig. 2). Only good quality radiographs were selected for pre and postoperative evaluation, including AP and lateral views with good exposure: having a uniform controlled distance of beam to the cassette was essential. Measurements were analysed by the Picture Archiving and Communication System (PACS), also known as Synapse (FUJIFILM Medical Systems Inc., Hanover Park, Illinois). All the radiographs were blinded from assessors by computerized random selection. Two independent orthopaedists were assigned to evaluate inter-observer and intra-observer reliability in radiographic measurement.

Our secondary outcome was the postoperative clinical results of patients who had AFC or SFC, including knee society score (KSS), functional score, knee ROM, and anterior knee pain at 6 weeks, 3 months, 6 months, 12 months and 24 months. We excluded staged bilateral TKA from clinical outcome evaluations to reduce the confounding effects of differing recovery protocols and operating intervals between sides. The outcome assessors were blinded to treatment groups during the study period.

### Statistical analysis

The measurements collected from radiographs and clinical results were analysed by descriptive statistics as means and standard deviations. The difference between sides in individual patients was evaluated by using independent simple *t*-test and proportional data were analysed by Fisher’s exact test.

Both inter-observer reliability and intra-observer reproducibility for pre and postoperative radiograph measurements among evaluators were calculated by using an intra-class correlation coefficient (ICC). For the inter-observer reliability, measurements were performed by two adult reconstruction fellows (AC, KB). For intra-observer reproducibility, measurements were performed twice with an interval of three weeks between.

## Results

**Fig. 3:**
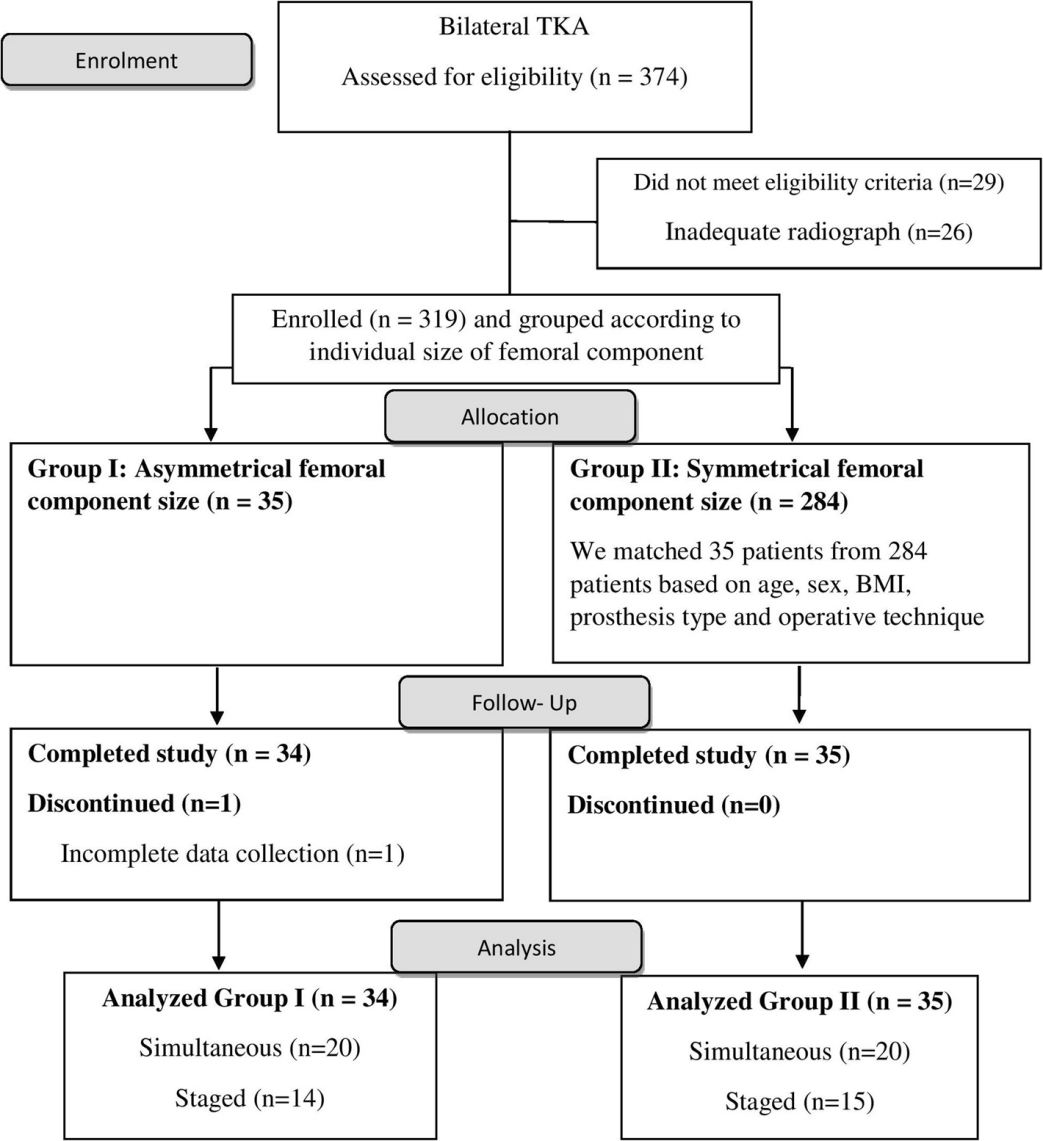
Flow diagram of the study. (TKA: Total knee arthroplasty, BMI: Body mass index)

A total of 374 patient notes were evaluated for entry (STROBE profile, Fig. 3). Fifty-five patients were excluded: 29 did not meet the ineligibility criteria and 26 had inadequate radiographs. We enrolled 319 patients and grouped them according to individual size of femoral components. Then, a retrospective matched pair (1:1) study of 70 patients who underwent bilateral TKA by a single surgeon; thirty-five patients with AFC size (Group I) and 35 patients with SFC size (Group II) were included. Only sixty-nine patients were analysed due to incomplete data collection for one patient who did not have a recorded KSS at 6 months after surgery in Group I. There were 20 patients of simultaneous bilateral TKA in each group. The incidence of AFC size in our study was 9.89% (37/374).

Preoperative demographic data are shown in Table 1. In Group I, 28 were female, and 6 were male, with an average age of 67.57 ± 8.9 years old; of the 35 patients in Group II, 27 were female, and 8 were male with an average age of 68.14 ± 6.8 years old. Patient demographics were not significantly different between the two groups. Preoperative radiographic data (Table 2) comparing the anatomy of both knees between Group I and Group II showed no outstanding discrepancies in AP femoral condyle diameters, AP femoral canal diameters, anterior and posterior femoral offsets, and ML diameter of femoral condyles.

**Table 1:** Demographic data for patients in the study

Characteristics	Group I	Group II	*P*-value
	Asymmetrical femoral component	Symmetrical femoral component group	
	(*n* = 34)	(*n* = 35)	
Gender (F/M)	28/6	27/8	0.76*
Age (years)	67.57 ± 8.9	68.14 ± 6.8	0.85^†^
BMI (kg/m^2^)	28.51 ± 3.4	28.04 ± 2.7	0.69^†^

*data analyses were performed with exact probability

^†^data analyses were performed with Student’s t-test

**Table 2:** Measured preoperative radiographic parameters for the left and right knees and their mean differences for Groups I and II

Parameter	Group I			Group II			*P* value^†^
	Asymmetrical femoral component			Symmetrical femoral component			
	(*n* = 34)			(*n* = 35)			
	Rt (mm)	Lt (mm)	Difference (Each patient)	Rt (mm)	Lt (mm)	Difference (Each patient)	
AP femoral canal diameter (A)	28.69 ± 2.55	28.57 ± 2.62	0.50 ± 0.46	28.65 ± 2.35	28.74 ± 2.41	0.53 ± 0.41	0.78
AP femoral condyle diameter (B)	64.34 ± 5.11	64.52 ± 4.62	2.11 ± 1.99	64.79 ± 4.67	65.06 ± 3.01	2.19 ± 1.69	0.87
Anterior femoral offset (C)	7.05 ± 1.75	7.37 ± 1.71	1.21 ± 0.95	6.67 ± 1.77	6.90 ± 1.86	1.16 ± 1.19	0.84
Posterior femoral offset (D)	27.19 ± 3.97	27.36 ± 4.14	2.14 ± 2.08	27.93 ± 4.30	27.57 ± 4.45	2.17 ± 2.31	0.95
ML femoral condyle diameter	74.88 ± 6.66	74.82 ± 6.00	1.03 ± 1.58	75.42 ± 5.41	75.45 ± 5.53	0.75 ± 0.94	0.38

^†^data analyses were performed with Student’s t-test

The postoperative radiograph (Table 3) noted a statistically significant (*p* = 0.007) imbalance in femoral component flexion of 2.18° ± 1.29° in Group I and 1.37° ± 1.08° in Group II. As the femoral component size was asymmetrical in Group I, the AP and ML femoral component diameters were significantly greater than those in Group II (*p* = 0.015 and *p* = 0.000, respectively). Group II had a slightly greater femoral valgus angle difference of the femoral component but was not statistically significant (1.64° ± 1.67°, and 1.41° ± 1.13°, *p* = 0.524). No statistically significant deviations in the other parameters (AP femoral canal diameter, anterior and posterior femoral component offset) were seen.

**Table 3:** Measured postoperative radiographic parameters in Groups I and II

Parameter	Group I	Group II	*P* value^†^
	Asymmetrical femoral component (Different each patient)	Symmetrical femoral component (Different each patient)	
AP femoral canal diameter (A’) (mm)	0.48 ± 0.44	0.60 ± 0.56	0.312
AP prosthesis diameter (B′) (mm)	2.33 ± 1.74	1.46 ± 1.05	0.015
Anterior femoral offset (C′) (mm)	1.13 ± 0.93	1.29 ± 0.99	0.500
Posterior femoral offset (D’) (mm)	2.23 ± 1.73	1.56 ± 1.12	0.066
ML prosthesis diameter (mm)	2.23 ± 1.41	0.72 ± 0.61	0.000
Femoral component flexion angle (8) (degrees)	2.18° ± 1.29°	1.36° ± 1.08°	0.007
Femoral component valgus angle (α) (degrees)	1.41° ± 1.13°	1.64° ± 1.67°	0.524

^†^data analyses were performed with Student’s t-test

For preoperative radiographs, the intra- and inter-observer kappa values (κ) were 0.69 and 0.63, respectively. For postoperative radiographs intra and inter observer kappa values (κ) were 0.72 and 0.64, respectively. Therefore, the reliability of the radiographic measurements was acceptable.

The postoperative clinical outcomes were measured only in the simultaneous bilateral TKA patients in each group to reduce the effect of confounding factors. No significant differences in postoperative clinical outcomes between the two groups were seen in the pre- and postoperative KSS, functional score, and knee ROM at 3 months, 6 months, 12 months and 24 months (Table 4). Of interest, both groups had the same incidence of anterior knee pain in each time period (Table 5).

**Table 4:** Postoperative clinical outcomes of bilateral simultaneous TKA between Groups I and II

Parameter	Group I	Group II	*P* value^†^
	Asymmetrical femoral component (*n* = 20)	Symmetrical femoral component (*n* = 20)	
Mean Knee Society Score			
Pre-op	53.32 ± 11.63	50.30 ± 13.84	0.29
6 weeks Post-op	78.55 ± 5.14	79.22 ± 4.55	0.53
3 months Post-op	86.82 ± 5.69	87.50 ± 5.15	0.57
6 months Post-op	91.95 ± 4.26	92.45 ± 4.16	0.59
12 months Post-op	92.10 ± 4.25	92.52 ± 4.08	0.65
24 months Post-op	92.17 ± 4.22	92.65 ± 4.02	0.60
Mean Functional Score			
Pre-op	45.5 ± 12.59	46.75 ± 10.03	0.62
6 weeks Post-op	60.75 ± 12.38	61.25 ± 8.71	0.88
3 months Post-op	73.25 ± 10.42	75.50 ± 8.25	0.45
6 months Post-op	84.50 ± 6.67	85.25 ± 6.78	0.72
12 months Post-op	84.25 ± 7.12	85.50 ± 6.47	0.56
24 months Post-op	84.75 ± 7.15	85.75 ± 7.12	0.66
Mean Maximum Knee Flexion Difference (Each patient)			
Pre-op	5.50° ± 4.68°	4.70° ± 4.49°	0.58
6 weeks Post-op	4.65° ± 3.76°	3.40° ± 3.59°	0.29
3 months Post-op	3.15° ± 2.85°	2.80° ± 2.67°	0.69
6 months Post-op	3.35° ± 4.04°	2.45° ± 2.82°	0.42
12 months Post-op	2.70° ± 3.23°	2.25° ± 2.17°	0.62
24 months Post-op	2.55° ± 3.02°	2.15° ± 1.93°	0.61

^†^data analyses were performed with Student’s t-test

**Table 5:** Incidence of anterior knee pain of bilateral simultaneous TKA between groups

Parameter	Asymmetrical femoral component size (yes/no)	Symmetrical femoral component size (yes/no)	*P*-value*
Preoperation	23 (13 patients)/17	15 (8 patients)/25	0.12
6 weeks postoperation	3 (2 patients)/37	5 (3 patients)/35	0.71
3 months postoperation	0/40	1 (1 patients)/39	1.00
6 months postoperation	0/40	1 (1 patients)/39	1.00
12 months postoperation	0/40	0/39	1.00
24 months postoperation	0/40	0/39	1.00

*data analyses were performed with exact probability

## Discussion

Asymmetrical component sizes in bilateral TKA have been studied extensively [8, 9]. However, clinical data have not been incorporated in determining the causes of bilateral asymmetry. In the study of Brown et al.*....*, of the 268 bilateral TKAs studied, there was a 6.7% size gap in femoral components between left and right knees, a 1.1% difference in tibial components, and a 0.3% distinction in patellar components [8]. Capeci et al evaluated 253 patients with simultaneous bilateral TKA and found 8.7%, 6.7% and 5.1% had femoral, tibial and patellar component asymmetry, respectively [9]. Reddy et al also reported 9.2% and 8.7% disparities in femoral and tibial component asymmetry within a total of 289 bilateral TKAs, respectively [10].

In our study, we noted just under 10% of our patients had AFC sizes following bilateral TKA that was performed by the same surgeon with the same surgical technique and prosthesis. Theoretically, asymmetry of bony anatomy and geometry could determine femoral component size selection, but this phenomenon has not yet been recorded in bilateral TKA patients. Yet, we observed no great variation in preoperative knee anatomy between the two groups. This implies that femoral bone geometry did not affect femoral component size selection for our patients (including AP and ML distal femoral bone geometry, anterior and posterior offset of distal femoral bone geometry). The imbalance between flexion and extension gap is one of the key factors that affected the femoral component size choice especially in the anterior refencing system where a bigger initial extension gap has typically resulted in surgeons selecting smaller femoral component size while a smaller initial extension gap has forced surgeons to choose the bigger femoral components to compensate for these gaps. However, in our study, we have particularly chosen femoral component size by using the “measure-resection bone cutting technique with posterior referencing system”; this creates the same amount of posterior femoral bone cut and flexion gap in all patients. Thus, any possible differences in femoral component size selection would not be affected by ligament laxity in our study.

Our study ascertained that the selection of the femoral component size was related to its angle of flexion and its coronal plane deviation. The difference in femoral component flexion could have resulted from different points of entry on the distal femur when the distal femoral cutting guide was inserted, bone cutting errors due to flexure of the thin cutting saw blade, forceful misdirection of the cutting saw blade, or movement of the cutting guide during osteotomy [12, 13]. A drill hole placement that is positioned too anteriorly generally leads to extension of the femoral component, whereas an excessive posterior drill hole placement might lead to flexion of the femoral component. An entry point deviation of just 5 mm anteriorly or posteriorly brought about a significant degree of flexion or extension (ranging from 2.2° of extension to 8.7° of flexion) [14]. Sagittal malalignment could create a 1-size-up or 1-size-down error in femoral sizing in TKA.

A computer simulation of TKA found that a femoral component flexion from 0° to 6° significantly resulted in smaller femoral size without changing flexion gap [15, 16]. Three dimensional imaging with prosthesis template software found that a 3° and a 5° extension of the distal femoral cut increased the AP femoral diameters by 2 and 3 mm, respectively, while a 3° and a 5° flexion decreased the AP femoral diameter by 2 and 3 mm, respectively [17]. Therefore, surgeons should carefully focus on the best distal femoral cutting angle to ensure an appropriate size selection of femoral component.

Theoretically, the component size would affect the functionality of the knee, but this was not borne out by our results. We found that the KSS, functional score and postoperative ROM were similar between AFC size and SFC size for patients; moreover, these findings are in agreement with previous studies [9, 10]. The similar postoperative ROM in both groups from our study could be due to the referencing system we used for femoral AP cuts. We used the posterior referencing system, which typically has the same posterior femoral bone thickness cut and does not affect the flexion gap. We had also anticipated differences in possible anterior knee pain which might have been due to the use of larger femoral component sizes. Larger components normally associated with increasing anterior offset, anterior overstuff, and patellofemoral contact force. Nonetheless, we did not see any of these outcomes in our study [5].

Different recovery of knee function (i.e. active ROM, quadriceps strength, and visual analogue scale pain score) between sides in simultaneous bilateral TKA has previously been examined and shown to be associated with risk factors such as female gender, old age, high BMI, high levels of anxiety, diagnostic differences, and different component sizes. We did not analyse these variables in our study; rather we focused on femoral component sizes [18].

Other limitations of our study were its retrospective design and small sample size which may not have completely represented the bilateral TKA population. We did not evaluate anterior bowing of femur from lateral radiographic view which may be a factor that influences femoral component size choice. All operations were performed by one surgeon with a specific type of prosthesis; therefore, decision making for component size choice will only reflect this particular surgeon’s experience, and the results are only generalizable to the same type of prosthesis. For the postoperative clinical outcome measures, our study utilized self-reported questionnaires which only represented the patients’ impression of their physical function. Performance-based tests such as the 2-min walking test and timed up and go tests may address other more objective aspects and complement these types of subjective questionnaires [19]. Furthermore, postoperative functional scores were included only in simultaneous bilateral TKA but not staged bilateral TKA because of the difficulty in interpreting data based on differing recovery protocols and confounding effects of different interval recovery times between the two sides.

## Conclusions

Our study showed that flexion of the femoral component and not preoperative bone anatomy determined the size of the AFC in patients undergoing bilateral TKA. Surgeons may want to be very careful to use a uniform cutting technique when performing distal femoral cutting during bilateral replacements as this affects the femoral component flexion and femoral size selection. The true lateral view of distal femur radiographs may assist surgeons in centralizing intramedullary drill guide into femoral canal and reduce the incidence of error in femoral flexion angle. In our study, AFC selection in bilateral TKA did not appear to greatly affect the outcome. Size selection remained independent of favourable clinical outcomes, including KSS, functional score, ROM in both asymmetrical and SFC size groups. Nonetheless, surgeons should still carefully and uniformly perform the distal femoral cutting as long-term studies need to be done on different prosthesis design and the outcomes of AFC size selection in bilateral TKA.

## Abbreviations

AFC: Asymmetrical femoral component
AP: Anteroposterior
BMI: Body mass index
ICC: Intra-class correlation coefficient
KSS: Knee society score
ML: Mediolateral
PACS: Picture archiving and communication system
ROM: Range of motion
SFC: Symmetrical femoral component
TKA: Total knee arthroplasty
